# A Highly Strained Phase in PbZr_0.2_Ti_0.8_O_3_ Films with Enhanced Ferroelectric Properties

**DOI:** 10.1002/advs.202003582

**Published:** 2021-02-18

**Authors:** Chuanwei Huang, Zhaolong Liao, Mingqiang Li, Changxin Guan, Fei Jin, Mao Ye, Xierong Zeng, Tianjin Zhang, Zuhuang Chen, Yajun Qi, Peng Gao, Lang Chen

**Affiliations:** ^1^ Shenzhen Key Laboratory of Special Functional Materials College of Materials Science and Engineering Shenzhen University Shenzhen 518060 China; ^2^ Electron Microscopy Laboratory, and International Center for Quantum Materials School of Physics Peking University Beijing 100871 China; ^3^ Department of Physics Southern University of Science and Technology Shenzhen Guangdong 518055 China; ^4^ Department of Materials Science and Engineering Hubei University Wuhan 430062 China; ^5^ School of Materials Science and Engineering Harbin Institute of Technology Shenzhen 518055 China

**Keywords:** Curie temperature, epitaxial ferroelectric films, highly strained tetragonal phase, negative thermal expansion

## Abstract

Although epitaxial strain imparted by lattice mismatch between a film and the underlying substrate has led to distinct structures and emergent functionalities, the discrete lattice parameters of limited substrates, combined with strain relaxations driven by film thickness, result in severe obstructions to subtly regulate electro‐elastic coupling properties in perovskite ferroelectric films. Here a practical and universal method to achieve highly strained phases with large tetragonal distortions in Pb‐based ferroelectric films through synergetic effects of moderately (≈1.0%) misfit strains and laser fluences during pulsed laser deposition process is demonstrated. The phase possesses unexpectedly large Poisson's ratio and negative thermal expansion, and concomitant enhancements of spontaneous polarization (≈100 µC cm^−2^) and Curie temperature (≈800 °C), 40% and 75% larger than that of bulk counterparts, respectively. This strategy efficiently circumvents the long‐standing issue of limited numbers of discrete substrates and enables continuous regulations of exploitable lattice states in functional oxide films with tightly elastic coupled performances beyond their present levels.

## Graphical abstract

A highly distorted tetragonal phase with giant negative thermal expansions is achieved in Pb‐based ferroelectric films via synergistic effects of misfit strainand laser fluence, which possesses considerable enhancements of spontaneous polarization (≈100 µC cm^−2^) and Curie temperature (≈800 °C). This strategy efficiently circumvents the issue of limited substrates and enables continuous regulations of ferroelectric films with tightly elastic coupled functionality.

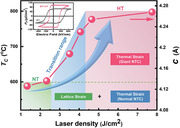

Electronic functional materials with broad operational temperature range and robust properties are of fundamental interest and technological importance for applications.^[^ ^1^, ^2^, ^3^ ^]^ Ferroelectric materials that can steadily resist polarization deterioration when operating at elevated temperatures are desirable as widely used in harsh environments. Recently, significant developments in thin film deposition technology have enabled high‐quality epitaxial ferroelectric films with novel crystalline/electronic structures and improved performances. Strain engineering, including lattice strain and thermal strain, has become one of the universally recognized approaches for extending new crystalline structures and intriguing elastic‐related physical properties in epitaxial functional films.^[^ ^4^, ^5^, ^6^ ^]^ In particular, coherent lattice strain could lead to consecutive structural transitions with tunable axial ratio (i.e., the ratio of the out‐of‐plane lattice to the in‐plane one, *c*/*a*),^[^ ^7^, ^8^ ^]^ enhanced Curie temperature (*T*_c_), and spontaneous polarization (*P*_s_) in epitaxial ferroelectric films.^[^ ^7^, ^9^, ^10^, ^11^ ^]^ Such an approach, although capable of yielding certain novel microstructures and emergent properties, is basically hampered due to the limited commercially single crystal substrates or film‐thickness‐driven strain relaxation.

To circumvent these challenges, alternative strategies reported have led to unusual structural states and enhanced properties in ferroelectrics.^[^ ^3^, ^12^, ^13^, ^14^, ^15^, ^16^, ^17^, ^18^ ^]^ Most intriguingly, a negative hydrostatic pressure applied on bulk PbTiO_3_ (PTO) could facilitate anomalous structural transitions, accompanying with simultaneous regulations of *c/a*, cell volume, *P*_s_ and *T*_c_.^[^ ^16^, ^17^ ^]^ Nevertheless, it is worth noting that the possibility of such manipulation in strained ferroelectric films is much more stringent, which is fundamentally restricted by the strain relaxation, or the shortage of sufficiently large misfit strain (*ɛ*) arising from limited substrates.^[^ ^17^ ^]^ In light of these, it is recently demonstrated that a combined control of epitaxial strains and growth‐induced defect dipoles could result in structural expansions (i.e., *c/a* and volume) in ferroelectric films.^[^ ^3^, ^18^ ^]^ It has been demonstrated, however, that the elongated *c/a* ratio barely affects the magnitude of spontaneous polarization *P*_s_, inconsistent with results previously reported.^[^ ^7^, ^17^ ^]^ Furthermore, ferroelectric materials with abnormal Poisson's ratio (PR, *v*) or thermal expansion (*α*), could perform very differently regarding their mechanical or thermal related functionalities.^[^ ^19^, ^20^ ^]^ In this circumstance, it has been shown that strained ferroelectric films with large *v* generally accompanies large *c/a* ratio and enhanced *P_s_*,^[^ ^7^, ^8^, ^21^, ^22^ ^]^ offering possibility in searching for strongly electro‐elastic coupled properties of functional materials.

By synergetic controllings of lattice strain, thermal strain and laser fluence during the film deposition, herein, we realize a highly strained tetragonal (HT) phase with increased *c*/*a* in a moderately strained [i.e., PbZr_0.2_Ti_0.8_O_3_/SrTiO_3_ (PZT(20/80)/STO), lattice strain *ε* = −1.2%] hetero‐structure system, accompanied by abnormally large negative thermal expansion (*α* = −1.9 × 10^−4^ / °C) and Poisson's ratios (*v* = 0.57). Meanwhile, the highly strained PZT(20/80) film exhibits prominent enhancements of both spontaneous polarization and Curie temperature, 40% and 75% larger than that of the bulk counterpart, respectively. Although the PZT(20/80) film is clamped with a fixed misfit strain, this strategy offers access to regulating distinct structural states (for instance, HT phase with large *c/a*) in a continuous manner, which provides a new pathway to effectively develop ferroelectric films with hidden phase and emergent electronic properties.

PZT(20/80) [*a*_(bulk)_ = *b*_(bulk)_ = 3.953 Å and *c*_(bulk)_ = 4.14 Å, *c*/*a* = 1.05, *T*_c(bulk)_ = 450 °C] films clamped by compressive misfit strains are generally accommodated by forming dislocations or/and complex domain patterns,^[^ ^23^, ^24^ ^]^ and exhibit a tetragonal phase with a slightly larger *c/a* ratio (i.e., *c/a =* 1.06).^[^ ^25^, ^26^ ^]^ In contrast to the conventional substrate‐induced lattice strain, here, a varying laser fluence during the pulsed laser deposition (PLD) process is employed as another significant factor to modulate structural and electronic properties of Pb‐based ferroelectric hetero‐structure systems. Intriguingly, PZT(20/80) films deposited with high laser fluence exhibit significant increases of the *c/a* ratio (from 1.06 to 1.1), remanent polarization (from 70 to 100 µC cm^−2^) and Curie temperature (from 600 to 800^ ^°C), although it is clamped with a small misfit strain (i.e., *ε* = −1.2%). **Figure** **1**a shows the laser‐fluence‐dependent structural transformations for ≈15 nm thick PZT(20/80)/STO hetero‐structure systems. Continuous shifts of the (002) diffraction peaks to lower angles indicate an elongating *c* lattice (from 4.13 to 4.27 Å), as the laser fluence is gradually increased (1.3 → 7.6 J cm^−2^). Relative to bulk counterpart, the *c* lattice is elongated up to 2.75% for the epitaxial PZT(20/80) films deposited with a laser fluence of 4.6 J cm^−2^, which is referred to as a highly strained tetragonal phase (HT, *c* > 4.2 Å). High‐resolution X‐ray symmetrical reciprocal space mappings (RSMs, in Figure 1b) further demonstrate a coherent growth for the PZT(20/80) films deposited with high laser fluences (> 4.6 J cm^−2^). In contrast, the PZT(20/80) films deposited under lower laser fluence (for instance, 1.3 J cm^−2^) are partially relaxed, which is consistent with the conventional normal tetragonal (NT, *c* = 4.13 Å) phase observed previously.^[^ ^25^ ^]^ Remarkably, the HT phase of PZT(20/80) films reaches an extremely tetragonality [i.e., (*c*‐*a/a*), ≈10%], which is about twice of bulk form (**Table** **1**). Meanwhile, an unexpected large PR (*v* = 0.57) is found in this newly found HT phase, in sharp contrast to that of the NT phase (≈0.33).^[^ ^27^ ^]^ This is a further proof that the HT phase exhibits highly distorted tetragonality and may contribute to enormously enhanced mechanical/elastic related properties. Figure 1c quantitatively reveals laser‐fluence‐driven *c* lattice and *c/a* ratio (≈1.1) for PZT(20/80) films. It also should be noted that the HT phase is thickness sensitive (Figure 1d). The pure HT phase can be sustained for thin (i.e., < 20 nm) PZT(20/80) films. An additional NT phase gradually arises and finally dominates for thicker PZT(20/80) films (for instance, 100 nm), according to the varying X‐ray diffraction (XRD) intensities of HT and NT phases, respectively. It is also revealed that the HT phase of PZT(20/80) films is tightly sensitive to the magnitude of misfit strain applied. No similar HT‐NT structural transition is achieved for PZT(20/80) films clamped with other lattice strains (i.e., other substrates) (see Figure S1, Supporting Information). In addition, there exists an analogous HT phase (Figure S2, Supporting Information) in slightly strained PbTiO_3_ hetero‐structure systems (i.e., PbTiO_3_/LSAT, *ɛ* = −0.8%), accompanying with an enormously enhanced tetragonality (≈10.55%) (Table 1). Thus, these observations indicate an universal existence of HT phase in Pb‐based ferroelectric films by leveraging the synergetic effect of misfit strain and laser fluence.

**Figure 1:**
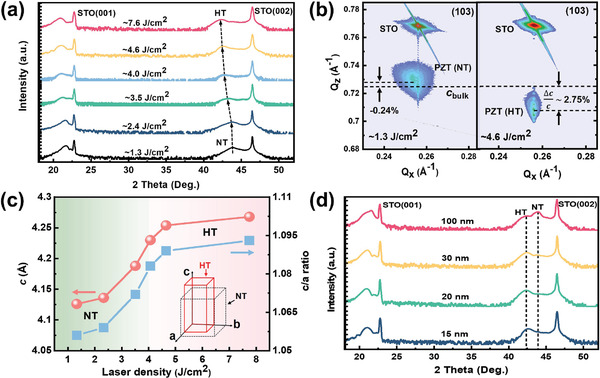
a) X‐ray diffractions of 15 nm thick PZT(20/80) films deposited with different laser fluence. b) Reciprocal space mappings of the PZT(20/80) films deposited with laser fluences of 1.3 and 4.6 J cm^−2^, respectively. c) The laser fluence dependent *c* lattice parameter and *c/a* of PZT(20/80) films. d) Thickness‐dependent structural transition in PZT(20/80)/STO hetero‐structure system grown with laser fluence of 4.6 J cm^−2^.

**Table 1:** Comparisons of lattice parameters and tetragonality [(*c*‐*a*)/*a*] of perovskite Pb‐based ferroelectric materials

Materials	*a* [Å]	*c* [Å]	((*c*‐*a*)/*a*) [%]
(HT) PZT(20/80)‐STO	3.905	4.27	9.35
(NT) PZT(20/80)‐STO	3.924	4.13	5.25
PZT(20/80) Bulk	3.953	4.14	4.73
(HT) PTO‐LSAT	3.868	4.276	10.55
(NT) PTO‐LSAT	3.868	4.138	6.98
PTO Bulk	3.899	4.15	6.44

Meanwhile, the stability of HT phase of PZT(20/80) films can be influenced by inserting a conductive SrRuO_3_(SRO) buffering layer. It is shown (**Figure** **2**a‐b) that the HT phase disappears after an bottom electrode (SrRuO_3_,SRO) inserted between the PZT(20/80) and the substrate [i.e., PZT(20/80)/SRO/STO]. In such circumstance, only a pure NT phase is observed in PZT(20/80) films larger than 15 nm. This is in sharp contrast to the mixed phases (i.e., NT & HT) in PZT(20/80)/STO hertero‐structure (Figure 1d) system. We conjecture that the disappearance of HT is mainly due to changes of electrical boundary conditions at the interface of PZT(20/80)/STO. Note that defect dipoles (i.e., charged point defects) will bring forth if ferroelectric films are grown with large laser fluences, which has been utilized to realize novel structure and functionalities.^[^ ^3^, ^28^ ^]^ To further verify the effect of charged defects driven by large laser fluences on HT phases, various electrical boundary conditions at the interface between PZT(20/80) film and substrates were examined. Of particular interest is that such effect is eliminated if the PZT(20/80) film is directly grown on an metallic SRO layer. This is consistent with our findings that only a pure NT phase was observed in the PZT(20/80)/SRO/STO system. It is, however, found that the HT phase reappears in the PZT(20/80)/STO/SRO/STO system if an insulating layer (STO, ≈1.0 nm) is inserted between the SRO and the PZT(20/80) film (Figure 2c‐d). Taken together, these findings highlight the critical role of the charged defects emerged during PLD depositions, which in turn, are mainly responsible for observed highly strained PZT(20/80) films with large lattice expansions. To get further insights into the microstructure and ferroelectricity of the HT phase, systematic high‐resolution scanning transmission electron microscopy (STEM) analysis is conducted to directly investigate the atomic‐scale lattice parameter and strain distribution. First, a low‐magnification STEM image (**Figure** **3a**) shows a clear PZT(20/80)/STO interface in an 80 nm thick PZT(20/80) film, which is deposited with a high laser fluence (i.e., 4.6 J cm^−2^). Figure 3b‐c is the atomically resolved high angle annular dark filed (HAADF)‐STEM images near the surface and the interface, respectively. The *c* lattice parameter near the surface from the HAADF‐STEM image (Figure 3b) is ≈4.15 Å. While the *c* lattice near the interface is notably elongated (≈4.35 Å), exhibiting an abnormally large tetragonality (*c/a* ≈1.1). Accordingly, the atomic‐level statistical results of *c* lattice evidently demonstrate that the 80 nm thick PZT(20/80) film consists of two distinct regions: the HT phase (*c* > 4.2 Å) with a thickness of ≈20 unit cells and the NT phase (*c* < 4.2 Å) for the rest of PZT(20/80) film. This is a further proof of coexisted NT & HT phases in PZT(20/80)/STO system, which is well consistent with the XRD results (as shown in Figure 1d). By contrast, only a pure NT phase (*c* < 4.2 Å) is observed in the 20 nm thick PZT(20/80)/SRO/STO system (see Figure S3, Supporting Information), reconfirming the effect of electrical boundary conditions (i.e., bottom electrode) on the existence of HT phase. Geometric phase analysis (GPA) is carried out (Figure 3f) to obtain detailed strain distributions in the PZT(20/80)/STO system. It is notable that the vertical strain (*ε*_zz_) of this PZT(20/80) film is inhomogeneous. In particular, *ε*_zz_ near the substrate is remarkably enhanced, corresponding to the HT phase with large lattice strain. By measuring the spatial variation of atomic displacement in the image plane of the Ti/Zr cation, we calculated the polarization *P*_s_ of the PZT(20/80) film with mixed HT& NT phases (Figure 3g). For the HT phase region, it is clearly seen that the *P*_s_ exhibits a remarkable enhancement (up to 140 µC cm^−2^), while the value of the NT phase (*c* < 4.2 Å) is comparable to that of bulk PZT(20/80) reported previously (≈70 µC cm^−2^).^[^ ^18^, ^29^, ^30^, ^31^, ^32^ ^]^

**Figure 2:**
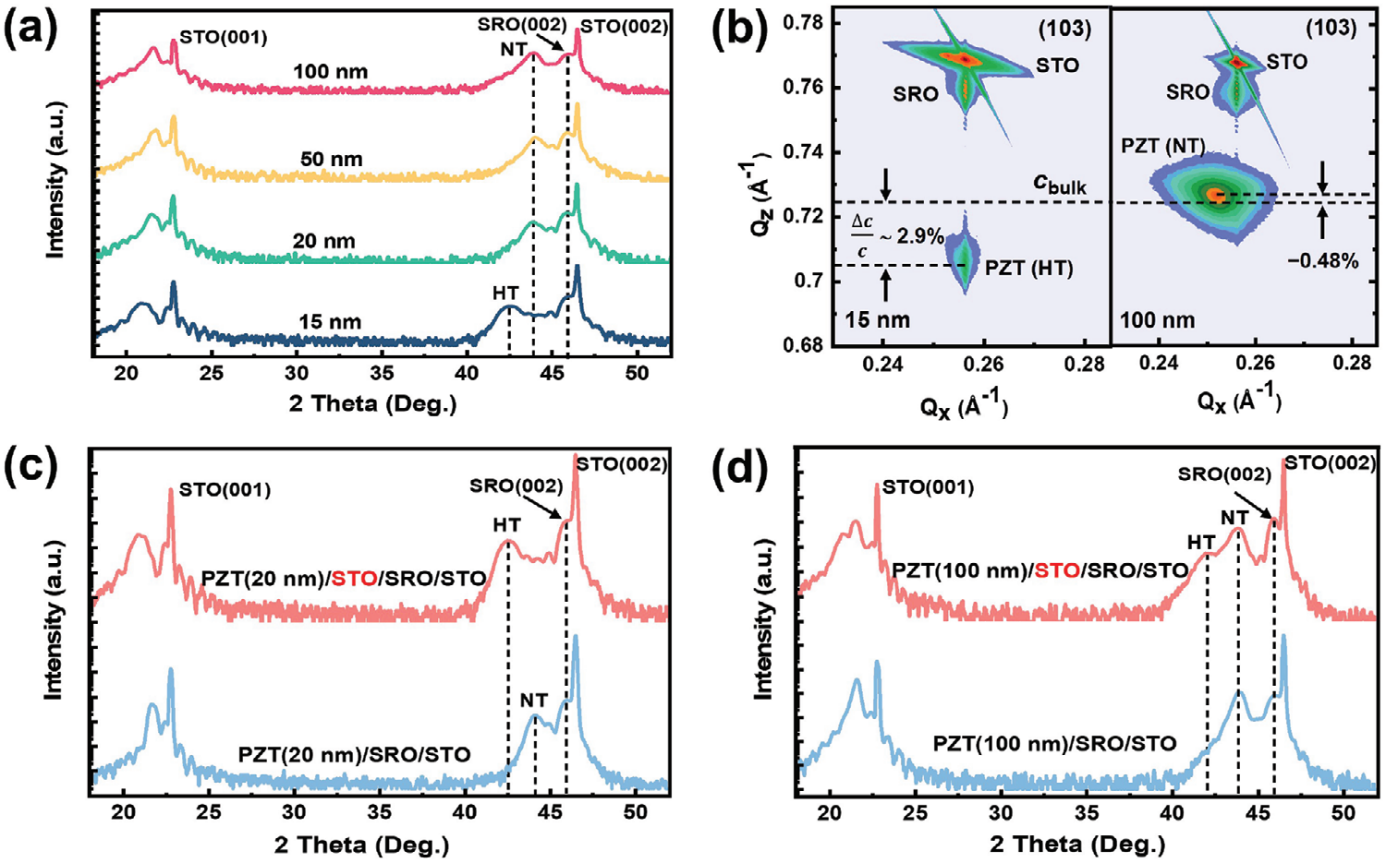
a) Film‐thickness‐dependent X‐ray diffractions of PZT(20/80)/SRO/STO systems deposited with the laser fluence of 4.6 J cm^−2^. b) Reciprocal space mappings of the corresponding PZT(20/80) hetero‐structure systems. c,d) Effect of a buffer layer (≈1 nm thick SrTiO_3_) on 20 and 100 nm thick PZT(20/80) hetero‐structure systems, respectively.

**Figure 3:**
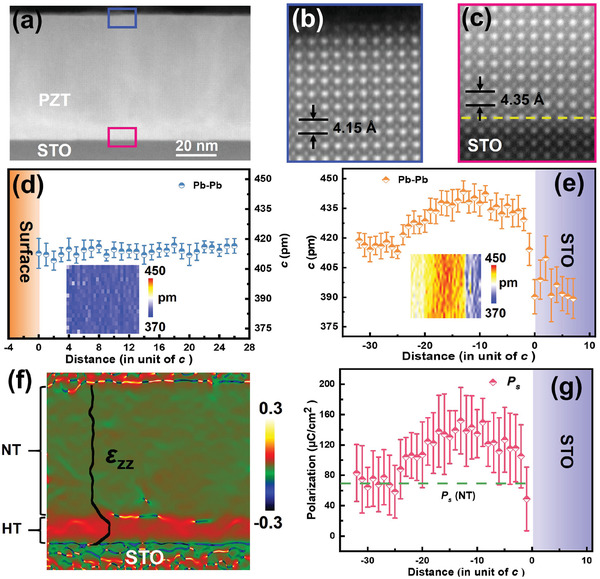
Atomic‐level analyses of the microstructure, strain distribution and spontaneous polarization of 80 nm thick PZT(20/80) film with mixed (NT & HT) phases. a) STEM image of the PZT(20/80)/STO system. b,c) The enlarged views of atomic structure near the surface and the interface, respectively. d,e) Lattice *c* near the surface and the interface. The inset is the 2D lattice parameter mapping of (*c*. f) Geometric phase analysis (GPA) of strain *ε*_zz_ for PZT(20/80)/STO system, and g) corresponding evolution of calculated spontaneous polarization as a function of the unit cell from STO to PZT(20/80) film.

Having established that PZT(20/80) films grown with large laser fluence possess an obviously elongated tetragonality, we now turn to investigate the switching property of ferroelectrics. Piezoelectric measurements (Figure S4, Supporting Information) were conducted in the PZT(20/80)/SRO/STO system with pure NT phase and PZT(20/80)/STO/SRO/STO system with mixed NT & HT phases. The results demonstrate flat surfaces (i.e., RMS ≈0.5 nm, 5 × 5 µm^2^) and switchable behaviors with apparent contrasts for both PZT(20/80) films. Furthermore, the well‐defined *P*–*E* hysteresis loops (**Figure** **4**a) reveal a desirable ferroelectricity for both PZT(20/80) systems. Remarkably, an unexpected large remnant polarization (*P*_r_) is found in the 100 nm thick PZT(20/80) films with mixed phases, far beyond the value (*P*_r_ ≈70 µC cm^‐^^2^) observed in the PZT(20/80) film with a pure NT phase. Meanwhile, the PZT(20/80) film with mixed phases exhibits a lower coercive field, relative to that of the pure NT phase. It is emphasized that the leakage (see Figure 4b) of the PZT(20/80) film with mixed phases is still acceptable, as asymmetrical bottom and top electrodes were considered. Thus, these systematic results clearly verify that the new‐found HT phase with elongated *c/a* ratio mainly contributes to the enhancement of *P*_r_, in well consistent with the result revealed through STEM observations.

**Figure 4:**
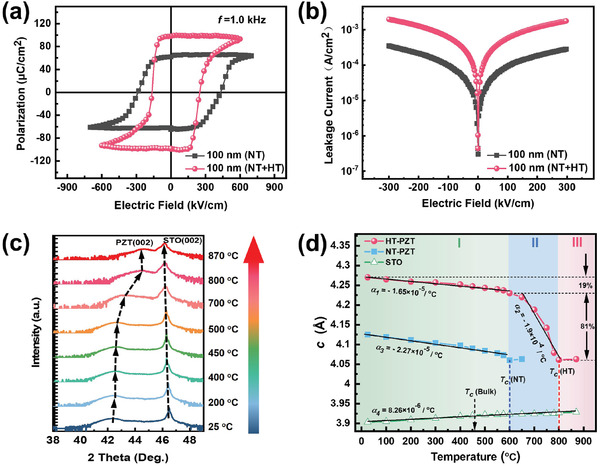
a) Ferroelectric hysteresis loops and b) corresponding leakage currents for the mixed (i.e., HT & NT) phase of PZT(20/80)/STO/SRO/STO system and the NT phase of PZT(20/80) /SRO/STO system, respectively. Temperature‐dependent XRD studies on c) 15 nm thick highly strained PZT(20/80) films and d) Temperature‐dependent *c* lattice and thermal expansions for 15 nm thick HT (*α_1_*&*α_2_*, circle), NT (*α_3_*, square) PZT(20/80) films, and the substrate STO (*α_4_*, triangle), respectively.

Ferroelectric materials generally show deteriorated polarization with increasing temperature.^[^ ^9^, ^33^ ^]^ Although the *T*_c_ of the known NT phase in strained PZT(20/80) films is increased (i.e., from 450 °C to 600 °C) via misfit strain alone,^[^ ^10^, ^33^, ^34^ ^]^ we herein obtain a further elevated *T*_c_ in the HT phase. As signaled by the temperature dependent lattice evolution (Figure 4c), the highly strained PZT(20/80) film can be persisted up to ≈800 °C. Figure 4d quantitatively compares the temperature‐dependent *c* lattices for both 15 nm thick PZT(20/80) films with a pure HT or NT phase. We remark on several distinct aspects of these curves: i) Above *T*_c_, the *c* lattice in all PZT(20/80) films is enlarged with higher temperatures, exhibiting a positive thermal coefficient (PTC). ii) Below *T*_c_ (i.e., 600 °C), the NT phase possesses a normal negative thermal expansion, with the coefficient (NTC, *α_3_* = −2.27 × 10^−5^ / °C) close to that reported in bulk ferroelectrics.^[^ ^35^ ^]^ iii) In contrast, the HT phase shows more intriguing thermal expansion behaviors. Its *c* lattice first contracts slightly (i.e., only 19%) over a wide temperature range (25–600 °C, Region I), with a similar normal NTC (*α_1_* = −1.65 × 10^−5^ / °C) to that of the NT phase (*α_3_* = −2.27 × 10^−5^ / °C). In contrast, the *c* lattice of HT phase in Region II (4.06 Å < *c* < 4.2 Å) experiences a pronounced decrease within the temperature range from 600 to 800 °C, regarded as a giant NTC (*α_2_* = −1.9 × 10^−4^ / °C). A close analysis of Figure 4d suggests that the giant NTC in region II dominantly contributes (as large as 81.0%) to the lattice expansion of HT phase, as compared to that of region I. To the best of our knowledge, this abnormal value is two orders of magnitude larger than that of previously reported perovskites with NTE.^[^ ^35^ ^]^ More intriguingly, this HT phase with giant NTC and abnormal large Poisson's ratio as discussed above, could behave very differently with respect to temperature or stress.^[^ ^36^ ^]^ On approaching 800 °C (Region III), the HT phase begins to expand with temperatures (i.e., PTC), accompanying with a transition from ferroelectric (tetragonal) phase to paraelectric (cubic) one.^[^ ^35^ ^]^ In addition, the HT phase exhibits extremely structural stability with time. No obvious shift of diffraction peak was observed in this highly strained PZT(20/80) films, even if stored at ambient atmosphere for 1 year (Figure S5, Supporting Information). These findings may help to develop thermal‐stable ferroelectric materials with promising applications operated at ultrahigh temperature environments.

To shed light on the structural regulation and enhanced ferroelectric properties of the highly strained PZT(20/80) film, time‐of‐flight secondary ion mass spectrometry (SIMS) was conducted to analyze the elemental composition for both NT and HT phases. Within the limits of experimental error, the SIMS results (Figure S6, Supporting Information) demonstrated that no obvious change of chemical content arises in the whole region of 100 nm thick PZT(20/80) film, although consisting of NT and HT phases. More particularly, there is no observable elemental inhomogeneity (for instance, Pb, Zr, and Ti) near the interface between HT/NT phases. Likewise, energy dispersive spectrometer analyses (Figure S7, Supporting Information) revealed homogenous elemental distributions between the mixed NT and HT phases of the PZT(20/80) film. We also found that oxygen post‐annealing of this highly strained PZT(20/80) film results in no change in crystalline structure and lattice parameters, eliminating the oxygen vacancy‐induced lattice expansion. Thus, we could exclude the impact of chemical nonstoichiometry on the occurrence of HT phase. Taking our findings together (**Figure** **5**), it is speculated that several effects cooperatively contribute to the unexpected HT phase with enlarged lattice parameter and resultant enhanced ferroelectric properties. As discussed above, charged defects emerged during PLD growth with large laser fluence first play a crucial role for forming HT phase. It is clearly seen from Figure 1 that larger laser fluences lead to expanded *c* lattice of PZT(20/80) film. Second, note that lattice‐mismatch induced strain at the interface is another vital factor to gain the new‐found HT phase. Systematical XRD measurements from Figure S1 reveal that the peculiar HT phase is strongly sensitive to lattice strains imposed from compressive to tensile substrates. Furthermore, the abnormally giant NTC observed in highly strained PZT(20/80) films additionally contributes striking lattice expansions. Along with the temperature decreasing, it is shown that the giant NTC (i.e., *α_2_* = −1.9 × 10^−4^ / °C) gives rise to rapid inflations of *c* lattices (region II in Figure 4d), as compared to that originated from normal NTC (i.e., region I). Accordingly, the giant NTC results in marked lattice expansions (up to 81.0%) in the HT phase of compressively strained PZT(20/80) films. However, further investigations should be highly demanded to verify the large laser fluence induced charged defects and resultant HT phase during the high‐temperature PLD deposition, which could offer another pathway to discover new structures and emergent properties in oxide films. Meanwhile, we found that there is a positive correlation between *c/a* ratio and ferroelectric properties of the present PZT(20/80) heterostructures, which is consistent with prior predictions in perovskite ferroelectric films.^[^ ^15^, ^29^ ^]^ In addition, as shown in Figure 3f, inhomogenous strains or complex defects are existed in HT phase, which could further boost the polarizations in Pb‐based oxide films.^[^ ^37^, ^38^ ^]^

**Figure 5:**
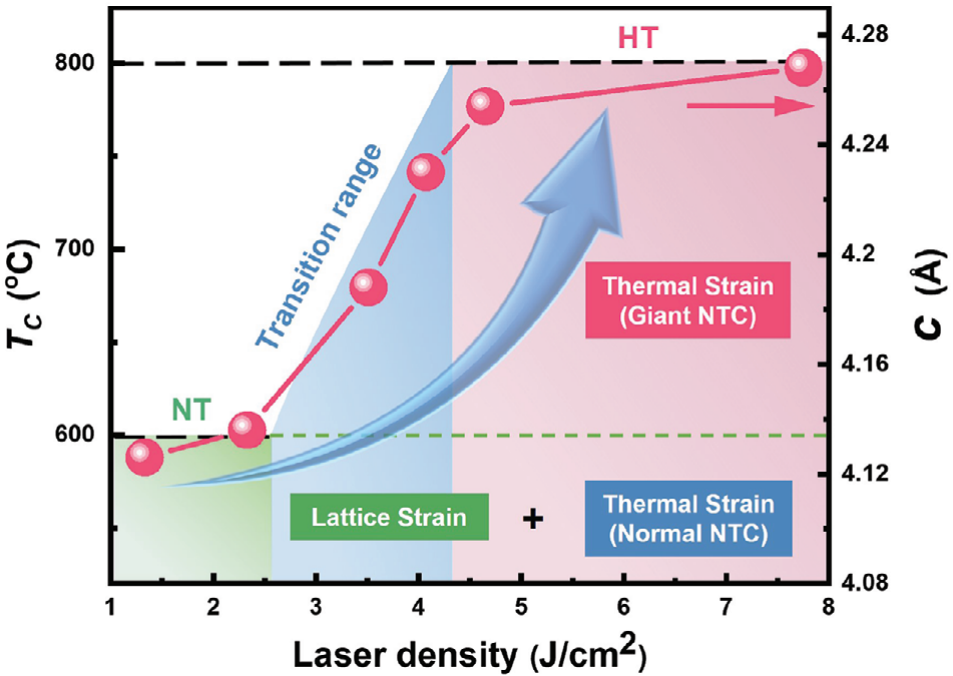
Constructed phase diagram showing laser fluence dependence of phase transformation, *c* lattice and Curie temperature of PZT(20/80) films.

In summary, we have demonstrated a highly distorted phase with abnormally giant mechanical/thermal coefficient (i.e., PR, NTC) in Pb‐based ferroelectric films, which is realized via synergistical effects associated with compressive misfit strain and large laser fluence. Concomitantly, more than 40% and 75% increases of the spontaneous polarization and Curie temperature were shown in the HT phase, compared to the values of PZT(20/80) reported to date. The advantages of the present strategy enable continuous modulations of unexplored structural lattices and electro‐elastic coupled physical properties in ferroelectric films,^[^ ^39^ ^]^ without the limitation of commercially discrete single‐crystal substrate. We also emphasize that the facile access to such large tetragonality could be widely applicable to other electronic oxide films with strong lattice–electron couplings, which offers great opportunities to extend the range of lattice control with exploitable phases and emergent functionalities.

## Experimental Section

##### Film Growth

15–100 nm PTO and PZT(20/80) films were grown via PLD using stoichiometric ceramic targets. The films were deposited in 13 Pa of oxygen pressure. The substrate was heated up to 600 °C. The laser energy density was varied by controlling the ablation spot size (measured using thermal paper) while maintaining the total incident energy. The spot size was adjusted through a size‐tunable optical grating located before the convex lens. The laser fluence was changed from 1.3 to 7.6 J cm^−2^. The 15 nm thick SRO bottom electrode was grown in 20 Pa of oxygen pressure. During the deposition, the substrate is fixed at 700 °C and the laser fluence is 1.5 J cm^−2^. All films were cooled down to room temperature at a cooling rate of 15 °C min^‐1^. The top electrodes (Pt, 20 × 20 µm^2^) were fabricated by a magnetron sputtering system. The area of the top electrodes was measured by an optical microscope.

##### Temperature‐Dependent XRD Analysis

The temperature‐dependent structure analysis was characterized by high‐resolution XRD (Smart Lab, Rigaku). The out‐of‐plane lattices of films were determined by *θ*−2*θ* scans, whereas the in‐plane lattice parameters and the epitaxy of the films were confirmed by RSM. The thicknesses of films were determined by X‐ray reflectivity measurements.

The cross‐sectional TEM samples were prepared by conventional mechanical polishing followed by argon ion milling in a Precision Ion Polishing System 691 (Gatan). The ion milling procedure consisted of two steps. In the first stage of coarse milling, the guns were at 4 keV with angles of 6° and −6°. In the following condition, the guns were set at 1 keV for 5 min with angles of 3° and −3° and were further lowered to 0.1 keV for 2 min for a final surface cleaning. HAADF‐STEM images used in this work were obtained from a probe Cs‐corrected FEI Titan 60‐300 (Titan Themis in Electron Microscopy Laboratory of Peking University) operated at 300 kV. Atom positions were determined by simultaneously fitting 2D Gaussian peaks to a perovskite unit cell using a home‐developed code running in MATLAB R2011a. Geometric phase analysis was performed using a free FRWRtools plugin for Digital Micrograph based on the original work by Hÿtch et al.^[^ ^40^ ^]^

Piezoelectric force microscopy (PFM) and atomic force microscopy (AFM) measurements were carried out via Asylum Research (MFP‐3D). Ferroelectric hysteresis loop was measured using a Radiant Tester (Precision LC II). To obtain direct depth profile of 100 nm thick PZT(20/80) film with mixed NT&HT phases, SIMS measurements are conducted. During the measurement, a cesium‐ion beam (1 keV) was employed to detect over a region of about 100 × 100 µm^2^. The signals of Pb, Zr, Sr, Ru, and Ti ions were recorded simultaneously along the film growth direction.

## Conflict of Interest

The authors declare no conflict of interest.

## Supporting information

Supporting Information
