# Supplementary material for: A Highly Strained Phase in PbZr0.2Ti0.8O3 Films with Enhanced Ferroelectric Properties
Source: Adv Sci (Weinh). 2021 Feb 18;8(8):2003582. doi: 10.1002/advs.202003582 (PMC8061395; doi:10.1002/advs.202003582)
Supplement: Supplementary file 1 — Supporting Information [file ADVS-8-2003582-s001.pdf]

WILEY-VCH

© 2020 Wiley-VCH GmbH

ADVANCED SCIENCE
Open Access

Supporting Information

for *Adv. Sci.,* DOI: 10.1002/advs.202003582

A Highly Strained Phase in $PbZr_{0.2}Ti_{0.8}O_3$ Films with Enhanced Ferroelectric Properties

Chuanwei Huang, Zhaolong Liao, Mingqiang Li, Changxin Guan, Fei Jin, Mao Ye, Xierong Zeng, Tianjin Zhang, Zuhuang Chen, Yajun Qi, Peng Gao,* and Lang Chen**

Supplementary Materials

A Highly Strained Phase in $PbZr_{0.2}Ti_{0.8}O_3$ Films with Enhanced Ferroelectric Properties

Chuanwei Huang, Zhaolong Liao, Mingqiang Li, Changxin Guan, Fei Jin, Mao Ye, Xierong Zeng, Tianjin Zhang, Zuhuang Chen, Yajun Qi,* Peng Gao,* and Lang Chen*

Dr.Chuanwei Huang, Zhaolong Liao, Changxin Guan, Fei Jin, Prof. Xierong Zeng
Shenzhen Key Laboratory of Special Functional Materials, College of Materials Science and Engineering, Shenzhen University, Shenzhen, 518060, China
E-mail: cwhuang@szu.edu.cn

Changxin Guan, Dr. Mao Ye, and Prof. L. Chen
Department of Physics, Southern University of Science and Technology, Shenzhen, 518055, Guangdong, China
Email:langchen@sustech.edu.cn

Changxin Guan, Prof.Tianjin Zhang, and Prof. Yajun Qi
Department of Materials Science and Engineering, Hubei University, Wuhan 430062, China
Email: yjqi@hubu.edu.cn

Dr. Mingqiang Li, and Prof. Peng Gao
Electron Microscopy Laboratory, and International Center for Quantum Materials, School of Physics, Peking University, Beijing 100871, China.
Email:p-gao@pku.edu.cn

Prof. Zuhuang Chen
School of Materials Science and Engineering, Harbin Institute of Technology, Shenzhen, 518055, China.

1. PZT(20/80) films deposited on different substrates

Apart from the influence from laser fluence, the emergence of the HT phase is strongly sensitive to the magnitude of susbtrate-induced misfit strain. It is shown that the HT phase ($c_{(PZT(20/80))}$= 4.25 Å) only emerges for PZT(20/80) films clamped with an appropriately compressive misfit strain (i.e., STO with ε = -1.2%). Although it is deposited with high laser fluences (i.e., ~7.6 J/cm^2), no HT phase arises for 15-nm-thick PZT(20/80) films when clamped with other substrates (i.e., LAO, LSAT, LGO, DSO and KTO). For example, the XRD results (**Figure S1**) show normal *c* lattices (ie., less than 4.2 Å) for these PZT(20/80) films deposited on KTO ($c_{(PZT(20/80))}$= 4.08 Å), DSO ($c_{(PZT(20/80))}$= 4.11 Å), LGO($c_{(PZT(20/80))}$= 4.16 Å) , LSAT ($c_{(PZT(20/80))}$= 4.02 Å) or LAO ($c_{(PZT(20/80))}$= 3.98 Å) substrate. In particular, no HT phase is formed for PZT(20/80) films deposited on a slightly larger (smaller) compressive misfit strain (LSAT with ε = - 2.2 % or DSO with ε = - 0.25 %), realtive to that deposited on STO (ε = - 1.2 %).

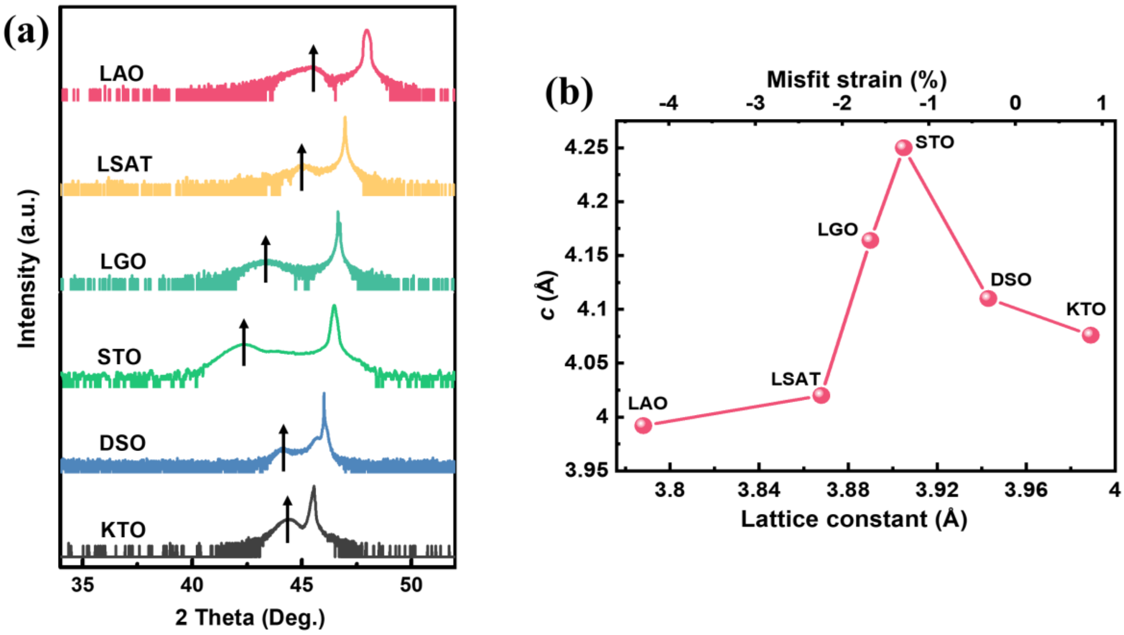

Figure S1. (a) XRD results of 15-nm-thick PZT(20/80) films grown on various substrates [$KTaO_3$(KTO), $DyScO_3$(DSO), $SrTiO_3$(STO), LGO($LaGaO_3$), $La_{0.3}Sr_{0.7}Al_{0.65}Ta_{0.35}O_3$(LSAT), and LAO($LaAlO_3$)], deposited with a high laser fluence of ~7.6 J/cm^2. (b) Corresponding changes of *c* lattice for PZT(20/80) films clamped by different misfit strains.

2. An analogous HT phase in PTO/LSAT hetero-structure system

Intriguingly, a comparable HT phase is realized in PTO hetero-structure system. By selecting an appropriate substrate (LSAT, ε = - 0.78 %), the HT phase turns up for PTO films deposited with large laser fluence. The PTO film possesses an extremely large *c* lattice (~ 4.276 Å) when it is grown at large laser fluences up to 5.0 J/cm^2 (**Figure S2**). The corresponding *c/a* (~ 1.106) is remarkably higher than that of bulk PTO (*a* = *b* = 3.899 Å, *c* = 4.15 Å, *c/a*=1.065).

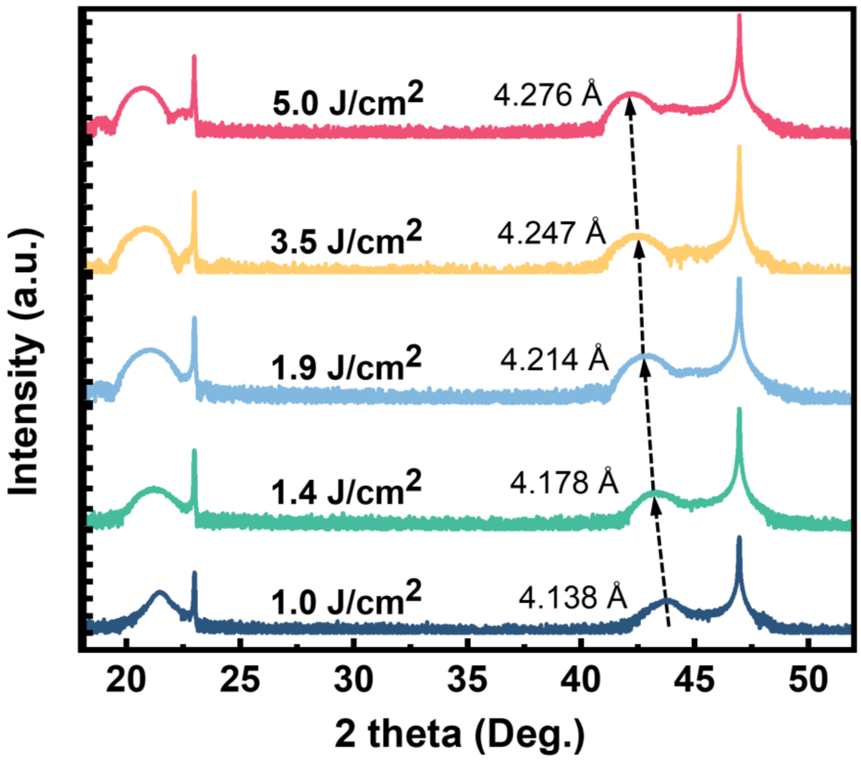

Figure S2. Laser fluence dependent XRD diffraction for 15-nm-thick PTO films grown on LSAT.

3. TEM images of the ~20-nm-thick PZT(20/80)/SRO/STO hetero-structure system

Atomic-level structures of the PZT(20/80)/SRO/STO system were studied via STEM. **Figure S(3a)** clearly displays sharp and flat interfaces. Meanwhile, the corresponding geometric phase analysis [**Figure S(3b)**] reveals a homogeneous *c* lattice strain distribution in the PZT(20/80) hetero-structure. **Figure S(3c)** directly demonstartes a pure normal tetragonal (NT) phase in the ~20-nm-thick PZT(20/80) film, where *c* lattice is less than 4.2 Å near the SRO region. This is a further proof that the electrical boundary condition (i.e., SRO) at the interface suppress the emergence of the HT phase.

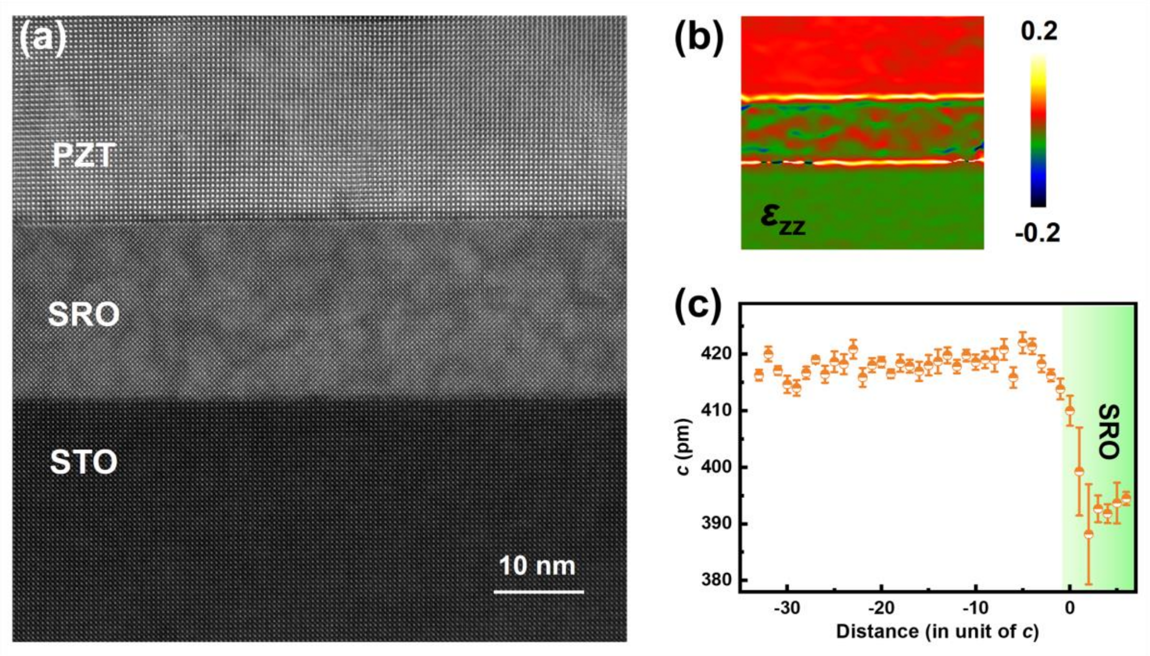

Figure S3. TEM images of the PZT(20/80)/SRO/STO hetero-structure system, demonstrating a normal tetragonal (NT) phase alone.

4. PFM characterizations in the PZT(20/80) hetero-structure system

Piezoelectric force microscopy (PFM) was conducted to check the topography and switch property of two100-nm-thick PZT(20/80) hetero-structure systems (**Figure S4**), consisting of the pure normal tetragonal [NT, **Figure S4(a-c**)] or the coexisted [NT&HT, **S4(d-f)**] phase. It is seen that both PZT(20/80) films exhibit an atomically flat surface [i.e., 5 μm × 5 μm, RMS ~ 0.5 nm shown in **Figure S(4a, 4d)**]. Furthermore, the out-of-plane PFM images [**Figures S4**(**b**, **c**, **e**, **f**)] clearly show switchable behaviors with apparent contrasts by applied electric fields (±7 V) in both PZT(20/80) films.

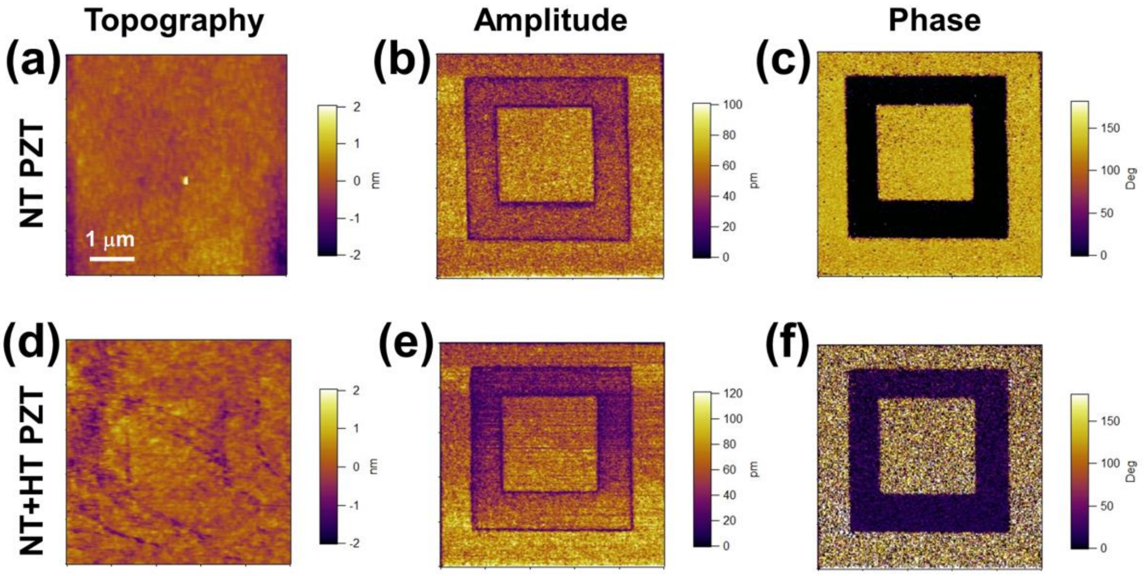

Figure S4. Topographies and switchable behaviors of the 100-nm-thick PZT(20/80) films with a pure NT phase (a-c) or mixed NT&HT (d-f) one.

5. Time-dependent structural stability of HT phase

It si shown that the phase exhibits an excellent stability over time. No obvious shift of XRD peaks (**Figure S5**) is observed in the 100-nm-thick PZT(20/80) film with mixed NT&HT phases, despite stored at the atmosphere for one year.

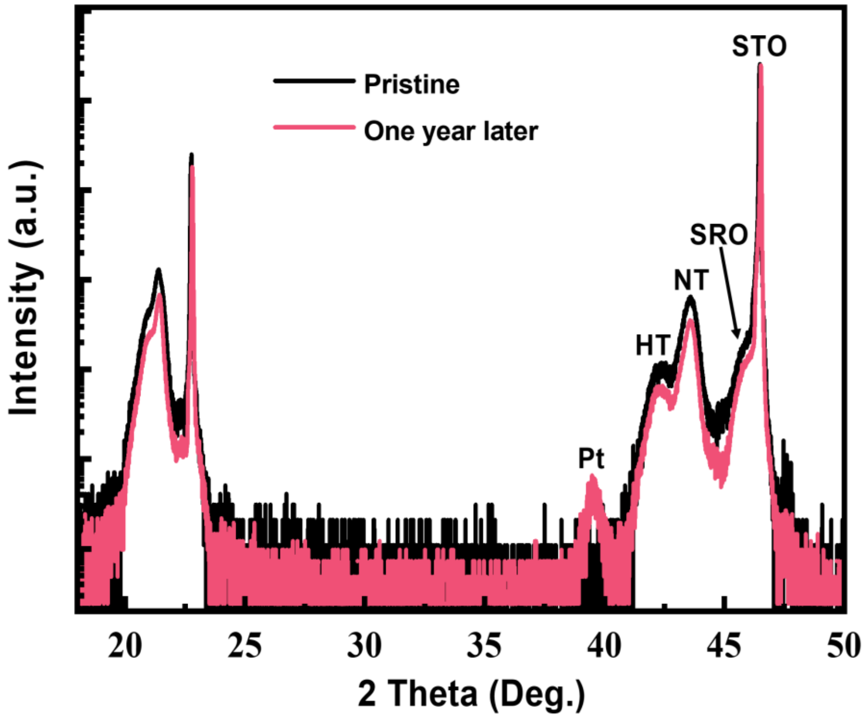

Figure S5. The stability of the HT phase of PZT(20/80) film over time.

6. The depth profile of cation chemistry in the mixed HT&NT PZT(20/80) film

To check the depth profile of cation stoichiometry, secondary-ion mass spectrometry (SIMS) was conducted. The SIMS results (**Figure S6**) reveal a nearly invariant chemical composition for the 100-nm-thick PZT(20/80) film with mixed NT&HT phases. Particularly, no sharp change of chemical compositions is emerged at the interface between the HT and NT phases. Thus, the high laser fluence induced chemical inhomogeneity could be ruled out, which is unlikely to result in the lattice expansion and enhanced ferroelectric properties observed in the highly strained PZT(20/80)/STO/SRO/STO hetero-structure system with a 1.0-nm-thick STO buffer layer.

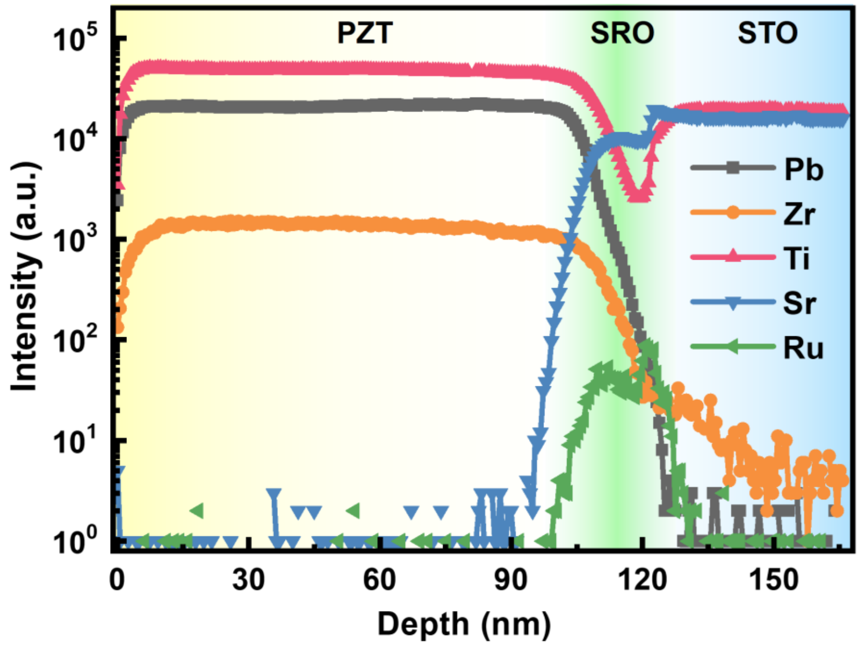

Figure S6. Elemental composition investigation of a 100-nm-thick PZT(20/80) film with mixed NT & HT phases.

7. The elemental distribution of PZT(20/80)/STO/SRO/STO hetero-structure system with a STO buffering layer.

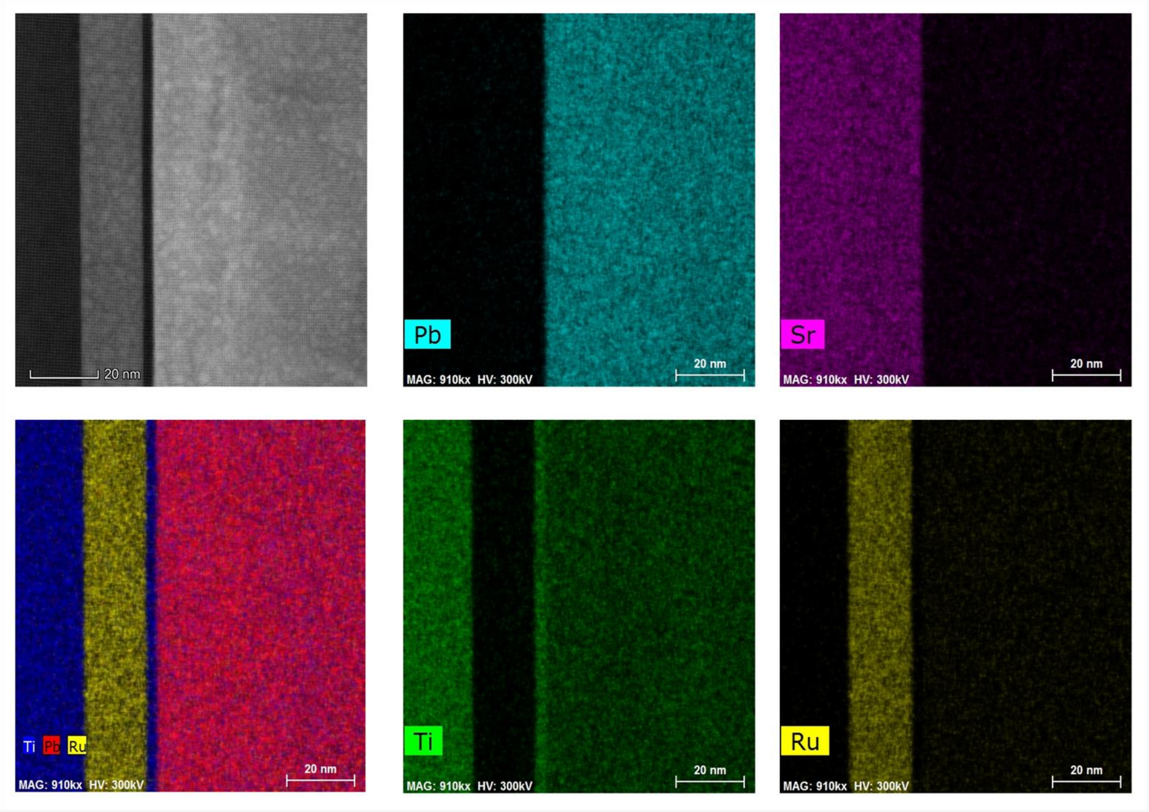

Figure S7. EDS analysis of a 100-nm-thick PZT(20/80)/STO/SRO/STO hetero-structure system with mixed NT & HT phases.
